# Clinical effect of bronchial arterial infusion chemotherapy and CalliSpheres drug‐eluting beads in patients with stage II–IV lung cancer: A prospective cohort study

**DOI:** 10.1111/1759-7714.13522

**Published:** 2020-06-30

**Authors:** Bin Shang, Jijun Li, Xingguang Wang, Daowei Li, Bin Liang, Yonggang Wang, Xujian Han, Weitao Dou, Gang Chen, Jianqiang Shang, Shujuan Jiang

**Affiliations:** ^1^ Department of Thoracic Surgery Shandong Provincial Hospital Affiliated to Shandong University Jinan China; ^2^ Shandong Medical Imaging Research Institute Affiliated to Shandong University Jinan China; ^3^ Department of Respiratory Shandong Provincial Hospital Affiliated to Shandong University Jinan China

**Keywords:** Bronchial arteries, chemoembolization, chemotherapy, lung cancer, microspheres

## Abstract

**Background:**

CalliSpheres are drug‐eluting beads used for tumor artery embolization, with clinical benefits in a number of cancer types. The aim of the study was to examine the clinical benefits and complications of patients with stage II–IV lung cancer treated with CalliSpheres drug‐eluting beads for embolization versus conventional vascular interventional treatment.

**Methods:**

This was a prospective cohort study conducted from August 2018 to May 2019. The patients were grouped according to traditional bronchial arterial infusion chemotherapy (infusion group) or bronchial arterial chemoembolization with CalliSpheres drug‐eluting beads loaded with adriamycin (CallisSphere group). Short‐term effects, serum tumor markers, and adverse reactions during follow‐up were compared between the two groups.

**Results:**

There were 60 participants enrolled into the study with 30 in each group including 54 men and six women, 42–78 years of age. In the CalliSphere group, compared with the infusion group, the disease control rate was 93.3% versus 73.3% (*P* = 0.080) and the objective remission rate (ORR) was 86.7% versus 60.0% (*P* = 0.039); the three‐ and six‐month progression‐free survival (PFS) and six‐month overall survival (OS) were better in the CalliSphere group (three‐month PFS: 96.7% vs. 73.3%, *P* = 0.026; six‐month PFS: 87.5% vs. 57.1%, *P* = 0.045; six‐month OS: 87.5% vs. 52.7%, *P* = 0.024); after treatment, the tumor markers in the CalliSphere group were lower (CEA: *P* < 0.001; CYFRA21‐1: *P* = 0.014). There were no differences in adverse reactions between the two groups.

**Conclusions:**

The clinical effect of bronchial arterial chemoembolization with drug‐eluting beads on lung cancer is probably significant and could improve the short‐term response, PFS, and OS in patients with stage IIIV lung cancer, without increasing severe adverse reactions.

**Key points:**

**Significant findings of the study:**

The clinical effect of bronchial arterial chemoembolization with drug‐eluting beads on lung cancer is probably significant and could improve the short‐term response, PFS, and OS in patients with stage II–IV lung cancer, without increasing severe adverse reactions.

**What this study adds:**

The ORR, PFS, OS was better in the CalliSphere group than that of infusion group; CEA and CYFRA21‐1 were significant lower in CalliSphere group.

## Introduction

Lung cancer is the most significant malignant tumor threatening human health and life in the world, with nearly 2.1 million new cases in the world every year,^1^, ^2^ 730 000 of them being in China.^3^ Non‐small cell lung cancers (NSCLC) are the most frequent (85%–90%) cause of malignant lung tumors, usually affecting adults who smoke and who are ≥65 years old.^4^ Tobacco use is the main risk factor.^5^ Other risk factors include environmental exposures and genetic predisposition.^5^

Due to the lack of effective screening and early diagnosis, most patients lose their opportunity of radical surgery, seriously affecting their prognosis.^5^ Therefore, a comprehensive multidisciplinary of lung cancer has become a focus of attention. Nonsurgical treatment of lung cancer mainly includes chemotherapy, radiotherapy, targeted therapy, immune therapy, and interventional therapy.^5^ Adverse reactions such as radiation pneumonitis, secondary bronchial stenosis, gastrointestinal reactions, bone marrow suppression, and immunological pneumonitis may reduce the patients' quality of life and affect their prognosis.^6^ Patients with interstitial fibrosis, advanced age, heart disease, emphysema, or pulmonary disease cannot tolerate standard treatments for lung cancer.^7^, ^8^ In addition, there is no effective treatment for patients with local tumor progression after initial chemotherapy and radiotherapy.^5^

Recently, interventional therapy has been increasingly applied in the treatment of stage II–IV lung cancer, due to its minimal invasiveness, effectiveness, easy tolerance, and few complications.^9^ Interventional therapy for lung cancer can be classified into vascular interventional therapy and nonvascular interventional therapy, according to the use of a vascular route. Bronchial arterial chemoembolization belongs to vascular interventions and can extend the action time of chemotherapy drugs specifically at tumor sites, increase drug concentration at the target site, and kill cancer cells more effectively.^10^ Embolization also leads to blocked arterial blood flow, leading to tumor ischemia and necrosis, and has an obvious hemostatic effect in patients with lung cancer and hemoptysis.^11^, ^12^

Drug‐eluting embolization beads are gradually being accepted in the clinic due to their characteristics of delivering chemotherapy drugs and permanently embolizing tumor blood vessels.^13^ CalliSphere is a recently introduced drug‐eluting embolization bead product that has shown promises in a variety of cancer types.^13^, ^14^, ^15^, ^16^, ^17^, ^18^, ^19^, ^20^, ^21^, ^22^, ^23^, ^24^, ^25^ Nevertheless, data about its benefits and complications still remain to be defined more adequately.

Therefore, the aim of the present study was to examine the clinical benefits and complications of patients with stage II–IV lung cancer treated with CalliSpheres drug‐eluting beads for embolization versus conventional vascular interventional treatment.

## Methods

### Study design

This was a prospective cohort study conducted from August 2018 to May 2019, and was approved by the Human Biomedical Research Ethics Committee of Shandong Provincial Hospital. Written informed consent was obtained from the patients.

### Participants

The inclusion criteria were as follows: (i) patients ≥18 years of age; (ii) patients with lung cancer diagnosed by biopsy^5^; (iii) patients with stage II–IV who were unable to tolerate or refused surgery, radiotherapy, or systemic chemotherapy, presented disease progression after systemic chemotherapy, or had airway compression due to a large tumor; (iv) patients with expected survival >3 months; (v) patients with ECOG ≤2; (vi) patients with white blood cells ≥3.0 × 10^9^/L, neutrophils ≥1.5 × 10^9^/L, hemoglobin ≥10.0 g/L, platelets ≥100 × 10^9^/L, and alanine aminotransferase, aspartate aminotransferase, bilirubin, and creatinine ≤1.5 times the upper limit of normal, normal coagulation, and normal blood urea nitrogen; (vii) patients with at least almost normal cardiopulmonary function; and (viii) patients who volunteered to participate in this study, signed the informed consent, had good compliance, and cooperated with follow‐up.

The exclusion criteria were: (i) contraindications to endovascular treatment and contrast agent; (ii) extensive and uncontrollable extrapulmonary metastatic lesions; (iii) cachexia or heart, lung, liver, or kidney failure; (iv) chemotherapy contraindications such as severe infection or severe thrombocytopenia; (v) contraindications to angiography such as serious bleeding tendency and iodine allergy; (vi) bronchial artery and spinal artery sharing the same trunk or with anastomosis in between, as detected by intraoperative angiography; (vii) other malignant tumors within five years; (viii) participating in another drug clinical trial within four weeks; (ix) diseases severely threatening patient's safety or influencing the patient's completion of this trial, as per investigator judgment; or (x) patients ineligible to be enrolled according to the investigator's judgment.

### Patient groups

The patients were grouped according to whether they underwent traditional bronchial arterial infusion chemotherapy (infusion group) or bronchial arterial chemoembolization with CalliSpheres drug‐eluting beads loaded with adriamycin (CallisSphere group). The treatment was selected based on a thorough discussion between the patient and the physician regarding the patient's physical and financial conditions. The patients were group‐matched by age, sex, cancer subtype, TNM stage, and number of metastases.

### Treatment: CalliSphere group

The patients underwent preoperative bronchial artery computed tomography angiography (CTA) to clarify the blood supply of the tumor. The affected bronchial artery was catheterized through the femoral artery to perform digital subtraction angiography (DSA) examination. After the blood supply artery of the tumor was confirmed, a coaxial microcatheter was inserted super‐selectively to the distal blood vessel. Then, 40–50 mg adriamycin was loaded into 2 mL (1000 mg/mL) CalliSpheres beads (100–300 μm; Hengrui Medical Co., Ltd., Suzhou, China). The adriamycin‐loaded beads were mixed with the contrast agent to obtain a 10 mL mixture. Approximately 3–4 mL of the mixture containing 600–800 mg beads was injected into each patient via the microcatheter to embolize the tumor blood supply. After the blood flow significantly slowed down, angiography confirmed that the tumor blood vessels and staining disappeared and stopped. If the tumor volume was too large to be covered by the 100–300 μm beads, embolization was completed using the 300–500 μm beads. In the case of multiple arteries feeding the tumor, each artery was treated as above.

### Treatment: Infusion group

The affected bronchial artery was catheterized through the femoral artery to perform a DSA examination. After the tumor blood supply artery was confirmed, chemotherapy (epirubicin/pirarubicin, 40/50 mg) was infused into the target artery.

In the infusion group, 12 patients received one session of treatment, 10 patients received two sessions, and eight patients received three sessions. In the CalliSphere group, nine patients received one session of treatment, 11 patients received two sessions, and 10 patients received three sessions. Whether or not to repeat the treatment was based on a CTA re‐examination and the physical condition of the patient at one month after each treatment. The criteria for a repeat treatment included: (i) regional enhancement in the tumor; (ii) visible bronchial arteries or collateral blood vessels supplying blood to the tumor area; and (iii) the patient had no contraindications.

### Observational indexes

Short‐term effect, serum tumor markers including carcinoembryonic antigen (CEA) and cytokeratin 19 fragment antigen (CYFRA21‐1) at seven days before and 30 ± 10 days after treatment and adverse reactions during follow‐up were compared between the two groups. The patients with normal levels of CEA and CYFRA21‐1 were excluded for analysis. The disease control rate (DCR) and objective remission rate (ORR) were evaluated at the six‐month follow‐up according to RECIST 1.1.^26^ DCR was the percentage of cases with remission. ORR was the proportion of patients whose tumor volumes have reduced to a predetermined value and were maintained for a minimum time limit. Progression‐free‐survival (PFS) and overall survival (OS) were described in the FDA's Guidance for Industry: Clinical Trial Endpoints for the Approval of Cancer Drugs and Biologics (2018, https://www.fda.gov/media/71195/download). PFS was the time from grouping until the onset of tumor development (any aspect) or death (for any reason). OS was the time from grouping until death (for any reason). The patients lost to follow‐up were excluded for further analysis. Adverse reactions were graded according to CTCAE 5.0.^27^

### Patient follow‐up

The patients were followed‐up at 30 ± 10 days, 3 months ±10 days, 6 months ±10 days, 1 year ±10 days, 1.5 years ±10 days, and 2 years ±10 days. Imaging and biochemical examinations were conducted during follow‐up.

### Statistical analysis

SPSS 24.0 (IBM, Armonk, NY, USA) was used to analyze the data. Continuous data were expressed as means ± standard deviations and tested using the Student *t*‐test. Categorical data were expressed as a percentage and analyzed with the chi‐square test. Two‐sided *P*‐values <0.05 were considered statistically significant.

## Results

### Patient characteristics

**Figure 1:**
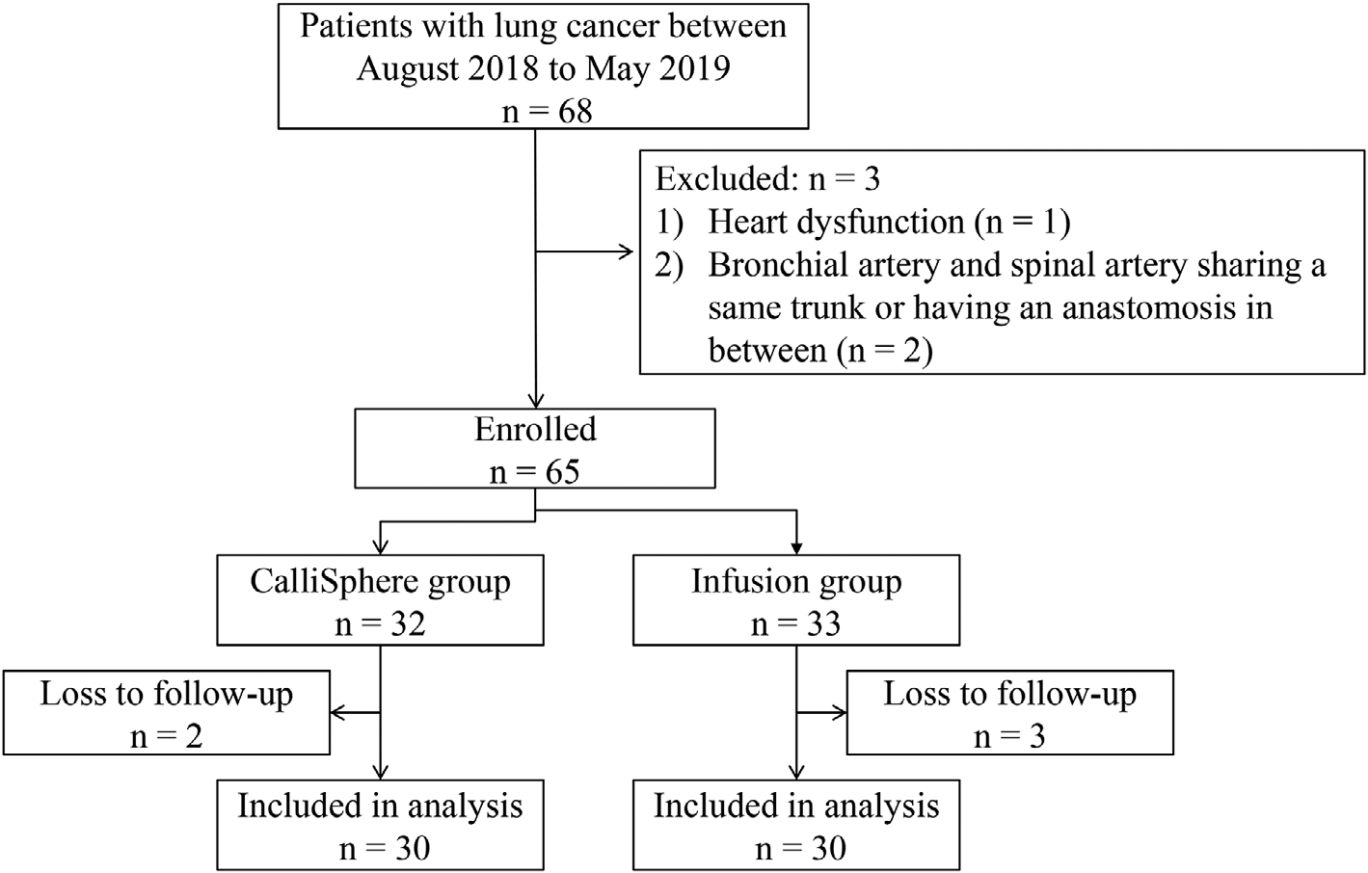
Patient enrollment flow chart.

**Figure 2:**
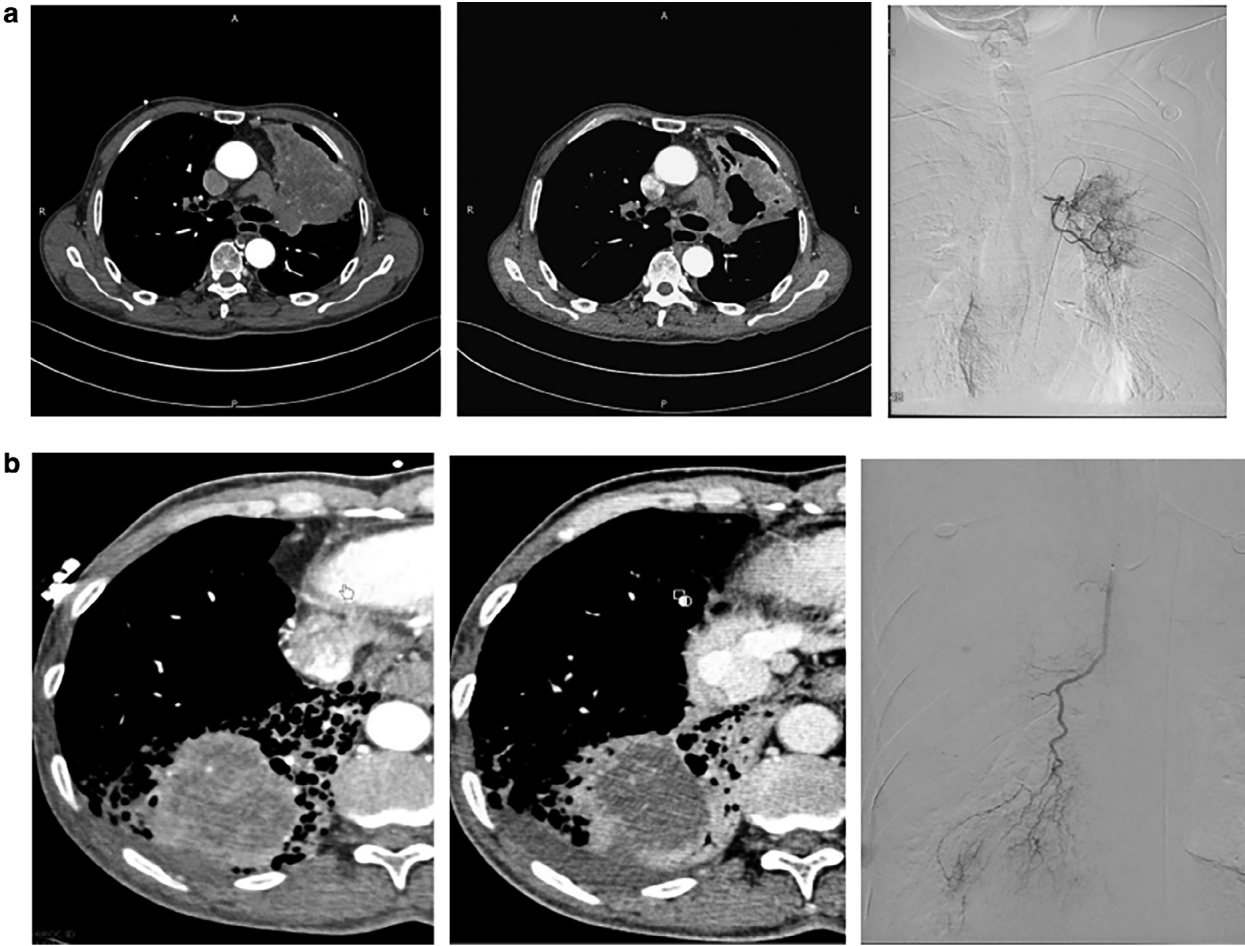
Representative images of computed tomography (CT) scan and intraoperative bronchial arteriography of the patients. (**a**) Patient 1: a 69‐year‐old male diagnosed with squamous cell lung carcinoma. Preoperative CT examination (left) showed a left hilar mass with enlarged mediastinal lymph node in the ascending branch of the left upper lobe. The CT re‐examination at one month after surgery (middle) showed tumor necrosis with cavity formation. Intraoperative bronchial arteriography (right) showed increased vascular branches in the left hilum and patchy tumor staining. (**b**) Patient 2: a 57‐year‐old male who was diagnosed with lung adenocarcinoma with mediastinal metastasis. Before surgery, the patient had received an esophageal stent implantation, followed by one session of radiotherapy and two sessions of chemotherapy. Preoperative CT (left) showed the tumor in the right lower lobe of the lung, accompanied by inflammation, atelectasis, and uneven enhancement. The CT re‐examination at one month after surgery (middle) demonstrated that the middle region of the tumor had no enhancement and showed low density. Intraoperative angiography (right) showed an increase in abnormal blood vessels in the right lower lung with tumor staining.

As shown in Fig 1, a total of 68 patients were screened, and 65 were enrolled in this study. Three patients were excluded due to heart dysfunction (*n* = 1), the bronchial artery and spinal artery sharing the same trunk, or having an anastomosis in between (*n* = 2). The enrolled patients were followed for a median period of 8.5 months (range, 2–16 months). After excluding the patients lost to follow‐up (two in the CalliSphere group; three in the infusion group), a total of 60 patients were included in the analysis (*n* = 30/group). These patients included 54 men and six women (42–78 years of age; mean, 65.9 years). The pathological types included squamous cell carcinoma (*n* = 42), adenocarcinoma (*n* = 16), and others (*n* = 2). Eight participants were stage II, eight were stage III, and 44 were stage IV. No statistically significant differences in age, gender, tissue type, tumor stage, metastasis, and tumor markers were found between the two groups (all *P* > 0.05) (Table 1). Representative images of CT and intraoperative bronchial arteriography of two patients in the CalliSphere group are shown in Fig 2.

**Table 1:** Patient characteristics

Characteristics	CalliSphere (*n* = 30)	Infusion (*n* = 30)	*P*‐value
Sex, n (%)			>0.999
Male	28 (93.3)	27 (90.0)	
Female	2 (6.7)	3 (10.0)	
Age (years), mean ± SD	66.1 ± 9.7	65.6 ± 7.5	0.868
Subtype, n (%)			0.883
Squamous cell carcinoma	22 (73.3)	20 (67.7)	
Adenocarcinoma	7 (23.3)	9 (30.0)	
Others	1 (3.3)	1 (3.3)	
TNM stage, n (%)			0.646
II	3 (10.0)	5 (16.7)	
III	5 (16.7)	3 (10.0)	
IV	22 (73.3)	22 (73.3)	
Number of metastases, n (%)			>0.999
≤3	21 (70.0)	22 (73.3)	
>3	9 (30.0)	8 (26.7)	

SD, standard deviation.

### Short‐term effect

As shown in Table 2, at the six‐month follow‐up in the Callisphere group, the DCR and ORR were 93.3% and 86.7%, respectively, while in the infusion group, the DCR and ORR were 73.3% and 60.0%, respectively (*P* = 0.08 for DCR and *P* = 0.039 for ORR).

**Table 2:** Therapeutic effect

Effects	CalliSphere (*n* = 30)	Infusion (*n* = 30)	*P*‐value
Complete response, n	0	0	0.060
Partial response, n	26	18	
Stable disease, n	2	4	
Progressive disease, n	2	8	
Disease control rate, n (%)	28 (93.3%)	22 (73.3%)	0.080
Objective response rate, n (%)	26 (86.7%)	18 (60.0%)	0.039

As shown in Table 3, the three‐ and six‐month PFS rates of the CalliSphere group were 96.7% and 87.5%, respectively, which were significantly higher than the 73.3% and 57.1% in the infusion group (*P* = 0.026 and *P* = 0.045, respectively). The three‐ and six‐month OS rates of the CalliSphere group were 96.7% and 87.5%, compared with 76.7% and 52.7% in the infusion group, but the difference was only significant at six months (*P* = 0.052 and *P* = 0.024, respectively).

**Table 3:** Comparison of short‐term effect

Survival rates	CalliSphere (*n* = 30)	Infusion (*n* = 30)	*P*‐value
3‐month PFS rate (%)	96.7	73.3	0.026
6‐month PFS rate (%)	87.5	57.1	0.045
3‐month OS rate (%)	96.7	76.7	0.052
6‐month OS rate (%)	87.5	52.4	0.024

PFS, progression‐free survival; OS, overall survival.

### Serum tumor markers

There were no significant differences in CEA and CYFRA21‐1 between the two groups before treatment (*P* > 0.05). After the treatment, the levels in the CalliSphere group were significantly lower than those in the infusion group (*P* < 0.001 and *P* = 0.014, respectively) (Table 4).

**Table 4:** Comparison of serum tumor markers

Tumor markers	CalliSphere (*n* = 16)	Infusion (*n* = 12)	*P*‐value
Pretreatment			
CEA (ng/mL)	247.32 ± 667.92	570.22 ± 872.78	0.172
CYFRA21‐1 (ng/mL)	40.79 ± 54.09	36.49 ± 37.12	0.581
Post‐treatment			
CEA (ng/mL)	34.44 ± 54.58	962.42 ± 1596.05	0.001
CYFRA21‐1 (ng/mL)	8.51 ± 4.14	79.92 ± 119.68	0.014

CEA, carcinoembryonic antigen; CYFRA21‐1, cytokeratin 19 fragment antigen.

### Adverse reactions

The main adverse reactions after intervention in the two groups included nausea, vomiting, cough, and chest pain, and fever. Severe complications, such as severe pain and spinal cord injury were not observed. No significant differences were observed in adverse reactions between the two groups, either when considering all grades or according to grade (all *P* > 0.05, data not shown).

## Discussion

CalliSpheres are drug‐eluting beads used for tumor artery embolization, with clinical benefits in a number of cancer types. Therefore, this study aimed to examine the clinical benefits and complications of patients with stage II–IV lung cancer treated with CalliSpheres drug‐eluting beads for embolization versus conventional vascular interventional treatment. The results of this prospective cohort study strongly suggest that the clinical effect of bronchial arterial chemoembolization with drug‐eluting beads on lung cancer is probably significant and could improve the short‐term response, PFS, and OS in patients with stage II–IV lung cancer, without increasing severe adverse reactions.

The results of this study showed that the application of drug‐eluting beads for arterial chemoembolization had good short‐term clinical effects in the treatment of patients with stage II–IV lung cancer. It apparently increased the ORR and prolonged PFS, without a higher occurrence of serious adverse reactions. Lung cancer generally infiltrates into, or around, the bronchial lumen, and the blood supply of the bronchial mucosa mainly depends on the bronchial artery. The existence of such anatomical relationships lays a foundation for bronchial arterial infusion chemotherapy and embolization for lung cancer.^10^, ^13^, ^28^ In bronchial arterial infusion chemotherapy, the drug will have a transient impact because the normal arterial blood flow will rapidly reduce the drug concentration at the target site, shortening its time of action. On the other hand, chemoembolization with drug‐eluting beads can increase drug action time and concentration, as well as blocking tumor blood supply, leading to ischemic necrosis of the tumor in addition to the cytotoxic effect of chemotherapy. In particular, for lung cancer accompanied by hemoptysis, it can not only control the progress of primary lesion but also completely stop bleeding.^12^, ^13^, ^29^ Such dual effects of this therapeutic method are also seen in other cancer types, such as hepatocellular carcinoma.^15^, ^16^, ^18^, ^19^, ^23^, ^24^, ^25^

From August 2010 to May 2014, Zhu *et al*.^28^ analyzed the clinical effect of bronchial artery infusion chemotherapy in 36 patients with lung cancer. The effective rate, one‐year survival rate, and two‐year survival rate were 72.2%, 75.4%, and 52.1%, respectively. In this previous study, the effective rate and one‐year survival rate were slightly higher than in the infusion group in the present study (60% and 66.7%, respectively). The possible reason is that all patients in the study by Zhu *et al*.^28^ were stage III, while the majority of the patients in the present study were stage IV. Therefore, tumor staging probably still has an impact on the benefits and prognosis after chemoembolization with chemotherapy‐eluting beads.

There are many investigations on patients with lung cancer treated with bronchial infusion chemotherapy, but only Bie *et al*.^13^ performed a study on six patients with NSCLC who were ineligible or refused standard treatment, but received CalliSpheres loading with gemcitabine for chemoembolization between May 2017 and December 2018. The results showed that the six‐ and 12‐month OS rates were 100.0% and 66.7%, respectively, while the median PFS was 8.0 months (4–23 months) and the median OS was 16.5 months (7–23 months) .^13^. The results of this study support those in our CalliSphere group (six‐ and 12‐month OS rates were 95.2% and 87.5%, and PFS was 8.8 months).

The study by Bie *et al*.^13^ provide useful data about the use of CalliSpheres, but they had no control group. In the present study, a control group was included, group‐matched for clinical characteristics, but the participants were not randomized, leading the way to probable biases. At present, there is no related trial examining the difference in efficacy and safety between the two methods. The present study and the study by Bie *et al*.^13^ reported no serious adverse reactions, but this will have to be confirmed. The present study is also limited by its small sample size and short follow‐up. Nevertheless, the present study lays the foundations for clinical trials of chemoembolization with chemotherapy‐eluting beads for the management of lung cancer.

In conclusion, this prospective cohort study strongly suggests that the clinical effect of bronchial arterial chemoembolization with drug‐eluting beads on lung cancer is probably significant and could improve the short‐term response, PFS, and OS in patients with stage II–IV lung cancer, without increasing severe adverse reactions. This will have to be confirmed in clinical trials.

## Disclosure

All authors declare that they have no competing interests.
